# Chloride Ions’ Penetration of Fly Ash and Ground Granulated Blast Furnace Slags-Based Alkali-Activated Mortars

**DOI:** 10.3390/ma14216583

**Published:** 2021-11-02

**Authors:** Patrycja Duży, Mateusz Sitarz, Marcin Adamczyk, Marta Choińska, Izabela Hager

**Affiliations:** 1 Faculty of Civil Engineering, Cracow University of Technology, 31-155 Kraków, Poland; mateusz.sitarz@pk.edu.pl (M.S.); marcin.adamczyk@pk.edu.pl (M.A.); izabela.hager@pk.edu.pl (I.H.); 2 IUT Saint-Nazaire, Research Institute in Civil and Mechanical Engineering GeM—UMR CNRS 6183, Nantes University, 44035 Nantes, France; marta.choinska@univ-nantes.fr

**Keywords:** alkali-activated materials, fly ash, chloride ions, gas permeability, water absorption

## Abstract

Due to the need to reduce the CO_2_ emissions of mineral binders, researchers are considering the use of alkali-activated materials (AAMs) as an alternative to cementitious binders. The properties of AAMs can be more advantageous than those presented by cementitious binders, and thus they can replace Portland cement binders in some applications. Mechanical tests of AAMs are being conducted on an ongoing basis; however, durability issues related to reinforcing steel in conditions in which steel members interact with chloride ions remain unsolved. In this paper, the precursors for AAM preparations are blends of fly ash (FA) and ground granulated blast-furnace slag (GGBFS) in four slag proportions: 0%, 10%, 30% and 50% expressed as a percent of FA mass. Four alkali-activated mortars were prepared, denominated as AAM 0, AAM 10, AAM 30 and AAM 50, respectively. Their basic physical and mechanical characteristics were investigated, as were their gas transport properties. The nitrogen Cembureau method was applied to determine the permeability of the mortar. The transport properties of the chloride ions were determined using the modified NT BUILD 492 migration test. The comparison of results obtained demonstrated a positive effect of GGBFS addition in terms of an increase in bulk density, permeability, porosity and, at the same time, a reduction in chloride ion penetration. The water absorption tests also provided insight into the open pore structures of mortars. The measurements revealed a strong dependence between fluid transport through the mortars and the water absorption and initial water content of materials.

## 1. Introduction

Alkali-activated materials (AAMs) are recognized as innovative substitutes to ordinary Portland cementitious binders. AAMs are systems capable of reducing the carbon dioxide pollution in the construction industry. The production of alkali-activated materials requires aluminosilicate precursors such as metakaolinite, or industrial waste materials such as fly ash (FA), ground granulated blast-furnace slag (GGBFS) or red muds. The utilization of waste streams is one of the numerous advantages of AAM applications. These materials, in conjunction with alkaline solution, have excellent binding properties, chemical durability [1,2], fire resistance [3,4] and many other features that may reduce the necessity of Portland cement concrete usage.

A prerequisite for introducing geopolymers into general use is a solid foundation of knowledge supported by experimental studies. Over the last few decades, the growing interest in AAM has provided a great deal of information on the applicability and properties of geopolymers. Well-developed theoretical knowledge [5] and experimental studies [6,7,8] are presented in the literature; however, there remains a lack of clear instructions for designing AAM mixes, uniform testing standards and requirements for the values of properties, such as those specified for cement concrete. Some of the test methods dedicated to cementitious mixtures can be applied directly to alkali-activated materials, for example, testing compressive and flexural tensile strength or determining the apparent chloride diffusion coefficient by bulk diffusion [9]. In practice, ASTM 1556 [9] is time-consuming; for this reason, it is preferable to determine resistance to chloride using accelerated methods such as ASTM C1202 Electrical Indication of Concrete’s Ability to Resist Chloride Ion Penetration [10] or NT BUILD 492 [11], based on an external potential application. Unfortunately, the electrical conductivity of geopolymers and AAM is much higher than that of cementitious materials, so a rapid chloride permeability test cannot be directly applied for AAM testing. According to the literature [6], a weaker voltage is selected when geopolymers and AAMs are tested.

In general, in AAMs and geopolymers, fluid penetration through the material occurs due to the presence of an open-pore system [12]; additionally, the transport mechanisms in these materials are similar to cementitious binders and have been well investigated [13,14,15].

An experimental study was performed to define and verify the material parameters that affect the chloride ion penetration of geopolymers and AAM mortars [7,16,17]. However, due to the variability of precursor compositions, AAM chloride transport properties and their chloride-binding capacities may differ significantly.

Owing to the high complexity of the chloride ion penetration of AA mortars, the investigation presented in this paper is intended to determine the chloride penetration and other transport properties associated with the ability of fluids and gasses to pass through material. Several publications on alkali-activated fly ash mortars with comparable compositions are available.

Investigations on the rheological, mechanical and physical properties of AAM were conducted by the authors in their recent publications [18,19]. A microstructure analysis was also conducted [20], thereby enabling a thorough analysis to be performed on the evolution of properties with time. In this study, the durability aspects are investigated.

The main novelty of the present study consists in the fact that designed materials are able to set at room temperature, with no need of thermal curing, which involves energy savings at the material manufacturing stage [4,8,18] The research proved that high-performance materials could be produced, showing an impressive mechanical performance reaching 63.0 MPa at 28 days. Although slag addition improves the mechanical performances of fly ash geopolymer mortar considerably, the durability aspect required investigations.

The main objective of the present study is it evaluates the permeability, chloride ions penetration and in this study only one parameter changes: the ground granulated blast furnace slag content. This adds to the parametrical study of geopolymer binders and contributes to developing knowledge in this field. The nitrogen Cembureau method was applied to determine the bulk density, permeability, porosity and, at the same time, a reduction in chloride ion penetration. The water absorption tests also provided insight into the open pore structures of mortars. The measurements revealed a strong dependence between fluid transport through the mortars and the materials’ water absorption and initial water content. It is worth noting that the transport properties of the chloride ions were determined using the modified NT BUILD 492 migration test. In this research, the following material properties were investigated: chloride penetration, permeability, porosity and effective porosity. Moreover, water content was evaluated, confirming the differences of interlinked porosity between the materials.

The porosity results for those materials were investigated in authors’ previous studies, and citation to those results were added into the text in order to provide more data for analysis. The detailed influence of GGBFS on porosity changes and microstructure (measured by mercury intrusion porosimetry) is presented by one of the paper co-authors in [20].

## 2. Tested Materials and Methods

The studies were performed on AAMs produced with Połaniec (Poland) power plant fly ash, blended with GGBFS from Ekocem (Poland). The oxide compositions of both precursors are provided below (Table 1 and Table 2).

Sodium silicate solution with a molar ratio of 1.7 was used as an activator for the alkali activation synthesis process. All mixtures were characterized by the constant ratio values listed below. In this description, ‘binder’ is the sum of the dry precursors (FA; GGBFS).

- Water to binder (w/b) = 0.30;
- Alkaline solution to binder = 0.45;
- Sand to binder = 1.50.

The density of the GGBFS was 2.9 g/cm^3^, and the fly ash density was 2.1 g/cm^3^. Quartz sand of density 2.65 g/cm^3^ was used in this study. The composition of the mortars varied with regard to the in four GGBFS proportions: 0%, 10%, 30% and 50% expressed as a percent of FA mass, and the mixes were designated as AAM0, AAM10, AAM30 and AAM50. The components of the blends are presented in Table 3 for 1 m^3^ of mortar.

Material mixing was conducted in accordance with the optimized procedure recommended by Davidovits [21]. The samples were prepared in cylindrical molds of 100 mm diameter and 200 mm height and compacted with the use of a shaking table. After one day of curing at room temperature (18 ± 2 °C), protected from water evaporation, the samples were removed from the molds and kept in laboratory conditions. The specimens were stored in ambient conditions for approximately one year. All of the experiments were performed on ≈50 mm-thick discs with a diameter of 100 mm, cut from the middle section of the cylinders and preconditioned in accordance with standard recommendations.

The compositions of mortars were selected on the basis of previous research on compressive strength and flexural tensile strength [4,8]. The results of the tests show that the addition of a higher content of GGBFS positively affected the mechanical properties. The highest values of compressive strength and flexural tensile strength were observed for a blend in which 50% of FA was substituted by GGBFS. The values of compressive and tensile strength for each blend were measured after 3, 14 and 28 days, and the results obtained in the previous study of [8] are presented in Table 4.

**Figure 1:**
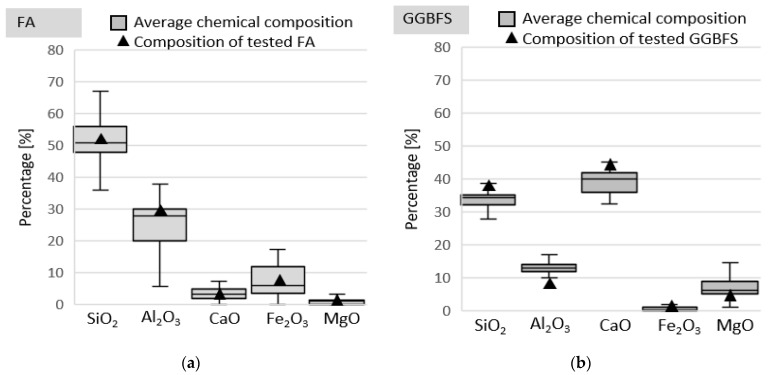
Comparison between the oxide compositions of tested precursors and the average values derived from reviewed studies: (**a**) fly ash and (**b**) ground granulated blast-furnace slag.

The transport of chloride ions in alkali-activated mortars is strongly affected by both the chemical and physical properties of the precursor (i.e., chemical composition, crystallinity and particle fineness) and also by the activator type. Moreover, the age and curing conditions may affect the transport properties of these materials. Figure 1 shows a comparison between the compositions of FA and GGBFS used in this study and the precursor oxide analysis data obtained by Osio-Norgaard, Gevaudan and Srubar [22] from studies on the chloride penetration of AAMs. This shows that the composition of FA and GGBFS remained typical for these types of aluminosilicate.

## 3. Test Methods

### 3.1. Physical and Transport Properties

The basic physical properties of the tested materials were specified: density, bulk density, total porosity and effective porosity (volume of open pores). This enabled the characterization of the pore system in order to establish the relationship between physical characteristics, permeability and chloride transport properties.

For the determination of bulk density, the dimensions and mass of the samples were evaluated. Density was determined by an inert gas (helium) injection in a gas pycnometer device.

The initial water content was also calculated in order to compare the capacity of the mortars to absorb water from the atmosphere when stored in ambient conditions. Water absorption tests were then conducted on the specimens, which were placed in a container with a gap between them and the bottom. The tank was filled with distilled water (temperature ≈ 20 °C) to a level of half the height of the specimen. After 24 h, the water level was increased to at least 20 mm above the samples. The specimens were stored in water until the mass was stabilized. The effective porosity was also calculated, which is defined in this study as the volume of pores that are available for water saturation, corresponding to the open porosity of the material.

### 3.2. Gas Permeability—The Nitrogen Cembureau Method

**Figure 2:**
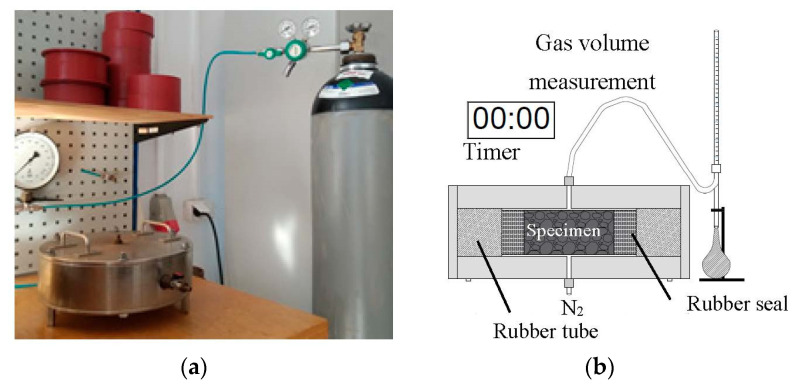
(**a**) Gas permeability cell; (**b**) scheme of gas permeability measurement.

The gas permeability of the AA mortars was determined by the RILEM–Cembureau method [23]. In accordance with tests described in the literature [7,13], permeability tests were performed under a lower inlet valve gas pressure (0.5, 1.0 and 2.0 MPa) than the Cembureau recommendation. The general principle is to apply a gas (N_2_) under a predefined pressure to a sample with a specific cross-sectional area and measure the flow rate (the time and volume of gas that passes through the sample) if the gas flow is stable. In the permeability cell, the sample is surrounded by a rubber tube with a minimum lateral pressure of 7 bar, ensuring gas flow only through the sample. The set-up is presented in Figure 2.

To compare the transport properties of AA mortars with different levels of water content in the material, the investigation was conducted on oven-dried (105 °C) disc samples of essentially constant mass, cooled down to the ambient temperature with no contact of moisture. The drying process, that preceded the permeability measurement was applied to the AAM samples that were one year old. For AAM and geopolymers when drying is applied, the temperature may trigger progress of polymerization [4]. The comparison between the permeabilities tested at dry reference conditions (dried at 105 °C) and for material that was stored at room conditions 18 °C and 75% RH. The results of the gas permeability measurements performed on the matching materials are presented in the authors’ previous research [8], and they will be used for comparison with the current experimental results.

### 3.3. Chloride Transport—Modified NT BUILD 492 Migration Test

**Figure 3:**
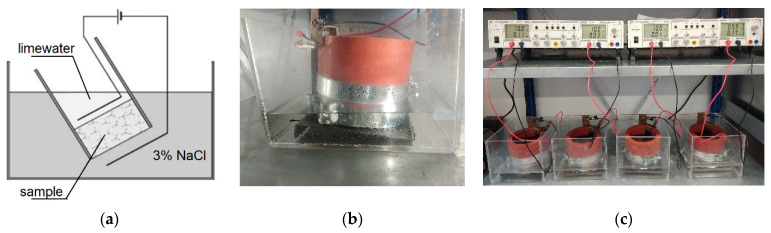
(**a**) NT BUILD 492 migration test scheme; (**b**) chloride migration cell; (**c**) chloride migration set-up.

The transport of chloride ions was evaluated using the NT BUILD 492 method [11]. The chloride migration coefficient was determined by the rate of the resistance of the material to chloride penetration. The samples were subjected to the chlorine environment after one year from casting. The test consists of applying an electrical potential axially across the specimen to enforce the movement of chloride ions from the outside to the inside of the sample. The schematic layout and set-up are presented in Figure 3.

**Figure 4:**
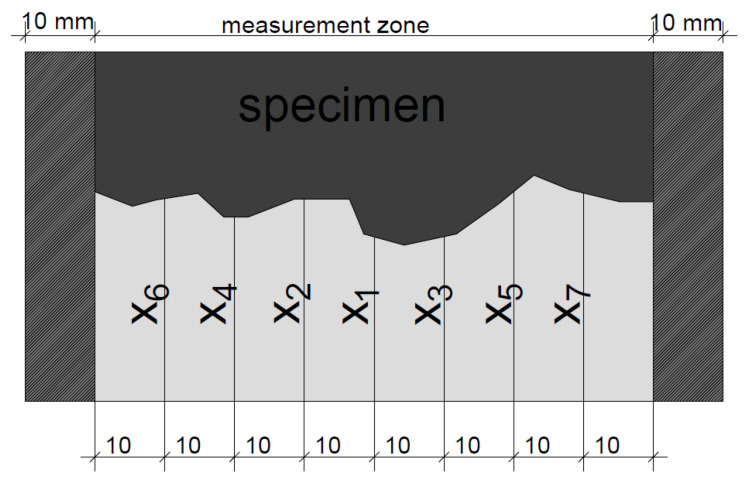
Principle of chloride penetration depth measurement.

After completion of the test, the specimen is axially split to enable the evaluation of the penetration depth. The average value of the penetration depth (x_d_) is measured after the use of the silver nitrate solution, as presented in Figure 4.

In reality, the procedure described in the NT BUILD 492 standard cannot be directly applied to AAM. Due to the geopolymers’ high electrical conductivity, the most reasonable solution to improve this testing method was to decrease the applied voltage from 60 V to 10 V, as recommended and experimentally validated for the ASTM 1202 method [6]. In view of the modification made to the test procedure, chloride transport properties were analyzed only by means of direct test results (penetration depth), excluding the chloride coefficient calculation. This approach provides an overview of the correlation between the mixture composition and other tested properties. Moreover, it enables us to identify the main trends and compare the resistance to chloride penetration for AA mortars with the changing content of GGBFS.

The tests were performed in two variants: 2.5 V applied for 18 h, and 10 V applied for 6 h. With reference to the Nordtest procedure, pre- and post-test temperatures of NaOH solutions were measured and the appropriate precision of measurements was ensured.

## 4. Results and Discussion

### 4.1. Physical Properties

**Figure 5:**
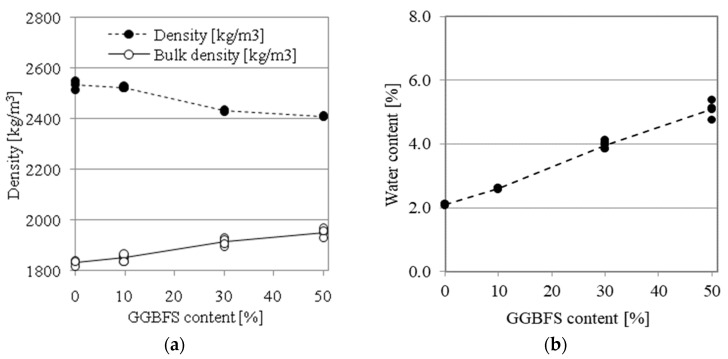
The effect of GGBFS content on the physical properties of AA mortars: (**a**) density; (**b**) initial water content.

The addition of GGBFS affects both the density and bulk density of tested AAMs. The results are presented in Figure 5a. The water content of mortars measured at age of one year from casting is presented in Figure 5b.

For mortars with higher GGBFS contents, denser mineral matrices were observed that prevented drying. The increasing value of bulk density for higher GGBFS content levels is a clear indication of structure densification, which coincides with higher mechanical performance. Moreover, the lower values of initial water content for lower GGBFS content levels indicate that the structure of the pore system enables easier water evaporation.

**Figure 6:**
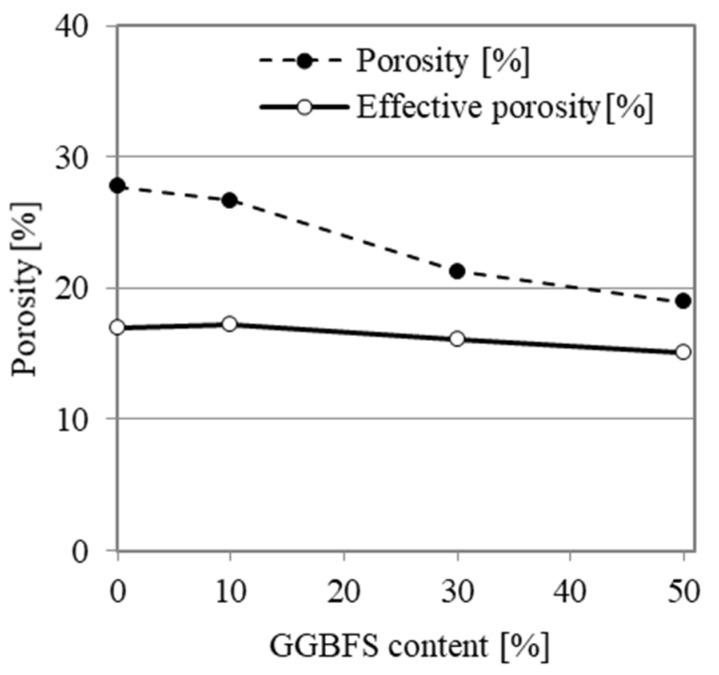
Porosity of AA mortars relative to GGBFS content.

Total porosity was calculated for oven-dried specimens as the ratio of bulk density to material density, expressed as a percent. The results are presented in Figure 6. The porosity values for AAM0, AAM10, AAM30 and AAM50 are 27.7%, 26.7%, 21.3% and 19.0%, respectively. The replacement of FA with GGBFS in the mortar composition caused a porosity reduction of 8.70 percentage points. While a significant decrease in the total pore volume was noted, the pore volume available for water saturation (effective porosity) decreased by only 1.9 percentage points, from 17.0% for AAM0 to 15.1% for AAM50.

### 4.2. Gas Permeability

**Figure 7:**
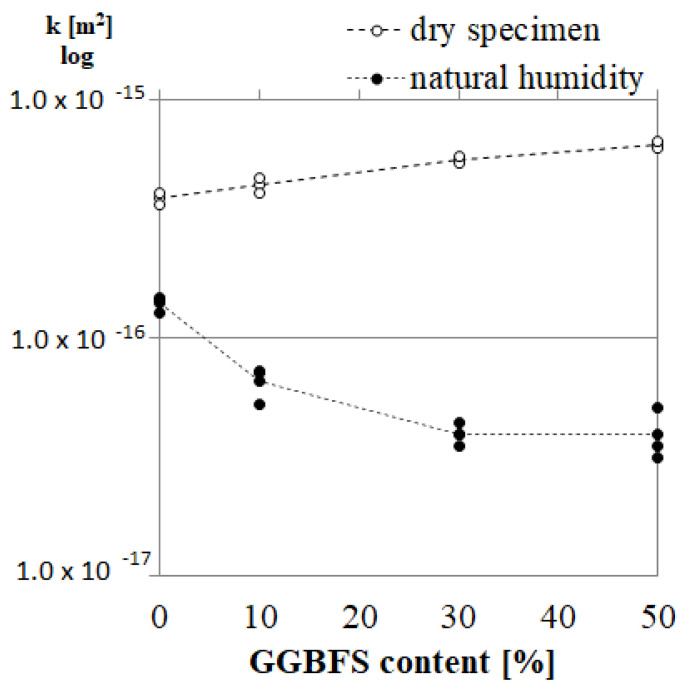
Permeability coefficient of AA mortars at ambient humidity and dry conditions relative to GGBFS content.

The gas permeability coefficient of AA mortars is depicted in Figure 7. The permeability measurements were performed on specimens stored in ambient conditions, in which the pores were partially filled with water (natural moisture content). The obtained results indicate a lower gas permeability with increasing GGBFS content. The average value of the permeability coefficient for AAM0 and AAM50 was 1.40 × 10^−16^ m^2^ and 3.92 × 10^−17^ m^2^, respectively. However, when the drying at 105 °C was applied, the values of permeability were not significantly different between the materials. Drying can be considered as a sort of heat treatment that may modify the porous structure, especially in cementitious materials where dehydration may occur. Although it is questionable but this drying procedure of mineral materials samples drying is frequently used when permeability of the material is evaluated with Cembureau method. The similar values of permeability of AAM after drying can be explained by the fact that the elevated temperature triggered a polymerization process that is more pronounced for the blends with lower GGBFS contents, AAM0 and AAM10. This effect was confirmed by research conducted by the authors in [4]. A temperature-induced process resulted in material densification and strength increase [4] in this research; the consequence was the permeability decrease for mortars with lower GGBFS content.

### 4.3. Chloride Penetration

**Figure 8:**
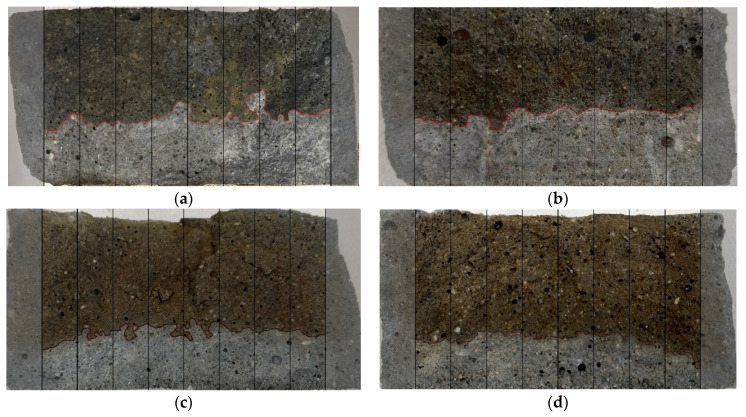
Specimens split for chloride penetration depth measurement after 18 h at 2.50 V: (**a**) AAM0 x_d_ = 21.20 mm; (**b**) AAM10 x_d_ = 20.92 mm; (**c**) AAM30 x_d_ = 17.33 mm; (**d**) AAM50 x_d_ = 14.04 mm.

**Figure 9:**
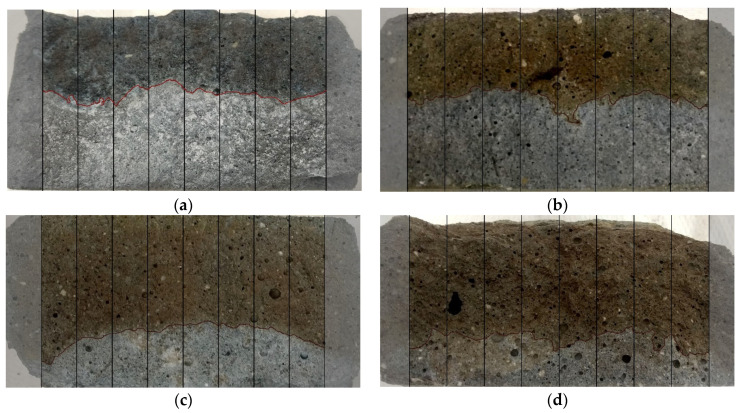
Specimens split for chloride penetration depth measurement after 6 h at 10 V: (**a**) AAM0 x_d_ = 26.80 mm; (**b**) AAM10 x_d_ = 25.03 mm; (**c**) AAM30 x_d_ = 17.75 mm; (**d**) AAM50 x_d_ = 13.46 mm.

Chloride penetration depth measurements were performed according to performance-based adjustments recommended in the literature [6]. The tests were performed in accordance with NT BUILD 492 [11] instructions for image analysis. The axial splits of specimens after an 18-h test and a 6-h test are presented in Figure 8 and Figure 9, respectively.

**Figure 10:**
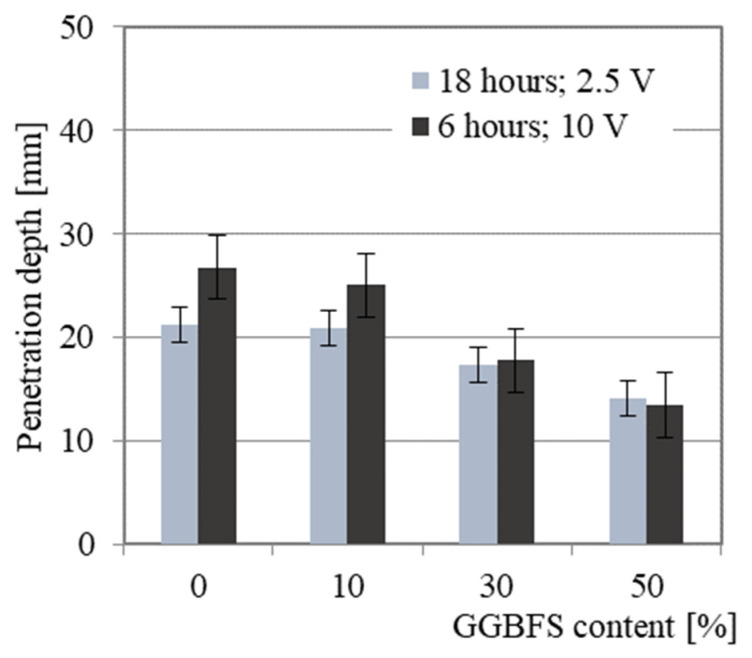
The average chloride penetration depths with reference to GGBFS content.

**Figure 11:**
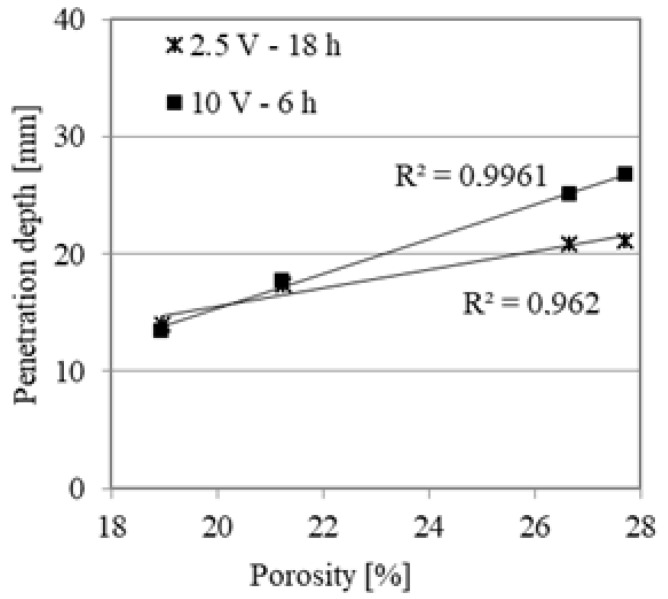
The average chloride penetration depths with reference to porosity.

The average chloride penetration depths for AA mortars are presented in Figure 10. As a general rule, increasing the GGBFS content results in lower chloride penetration. It is worth noting that the high electrical conductivity of geopolymers required the measurements to be performed at 10 V for 6 h. This test resulted in the following penetration depths: 26.80 mm, 25.03 mm, 17.75 mm and 13.46 mm for AAM0, AAM10, AAM30 and AAM50, respectively. For the 18-h test at 2.5 V, the results confirm the trend of decreasing chloride penetration depth with increasing GGBFS content. The material performances are closely related to porosity. The almost linear relationship between the penetration depth and porosity was found and is presented in Figure 11. Moreover, the addition at least 30% GGBFS to the mortar mixture results in consistently similar values for both tests, regardless of the conditions.

## 5. Conclusions

The conducted studies show that the addition of GGBFS to FA-based alkali-activated mortars has a substantial impact on the chloride penetration resistance and on other material properties such as permeability and effective porosity, which are strongly related to the penetration of chloride ions. The reported results indicate that blends of GGBFS with FA mortar compositions enable the manufacture of material with satisfactory mechanical and physical properties, with no additional thermal curing.

The following observations are noted:

- The moisture content of the samples stored in ambient conditions is higher for AAMs with a higher GGBFS content;
- Gas permeability is strongly dependent on the water content of the test specimen. For the specimens stored in ambient humidity a reduction in permeability with higher GGBFS content was observed;
- For oven-dried samples, the results of permeability do not differ significantly along with the GGBFS content. It is possible that the geopolymerization process was triggered by heat and for materials with lower GGBFS contents. Therefore, it is not recommended to use this drying procedure in AAM investigations;
- The addition of GGBFS reduces total porosity and, to a lesser extent, effective porosity (defined as the volume of pores that are available for water saturation);
- A linear relationship between the penetration depth and porosity was found;
- Chloride ion penetration is reduced by blending FS with GGBFS, resulting in a positive effect on the overall durability of the AAM.
- The effect of the addition of GGBFS on the chloride penetration rate shows a similar trend as that of changes in total porosity and effective porosity.
- The modified NT BUILD 492 uses an initial current voltage of 10 V instead of 60 V, allowing research to be performed on AAMs. The addition of at least 30% GGBFS reduces the sensitivity of the samples in the various tested conditions and provides a reliable evaluation of chloride ion penetration.

Further studies are required in order to adapt the testing procedures for AAMs and geopolymers, including the saturation of specimens, test duration and voltage, improving the accuracy of measurements.

## Figures and Tables

**Table 1:** XRF analysis of fly ash (FA).

SiO_2_	Al_2_O_3_	Fe_2_O_3_	CaO	MgO	SO_3_	K_2_O	Na_2_O	P_2_O_5_	TiO_2_	Mn_3_O_4_
52.30	28.05	6.32	3.05	1.71	0.28	2.51	0.76	0.69	1.35	0.07

**Table 2:** Composition of ground blast furnace slag (GGBFS).

SiO_2_	Al_2_O_3_	Fe_2_O_3_	CaO	MgO	SO_3_	K_2_O	Na_2_O	Cl¯	Na_2_Oeq	Blaine
39.31	7.61	1.49	43.90	4.15	0.51	0.356	0.468	0.038	0.702	3904

**Table 3:** AA mortar mix designs for 1 m^3^.

Components	AAM0 (kg/m³)	AAM10 (kg/m³)	AAM30 (kg/m³)	AAM50 (kg/m³)
Alkaline solution	330.70	333.90	340.60	347.50
FA	734.90	667.90	529.80	386.10
GGBFS	0.00	74.20	227.10	386.10
Sand (0/2 mm)	1102.42	1113.2	1135.30	1158.30

**Table 4:** Average values of compressive and flexural tensile strength for AAM [8].

Blend	Compressive Strength (MPa)			Flexural Tensile Strength (MPa)		
	3 Days	14 Days	28 Days	3 Days	14 Days	28 Days
AAM10	14.5	30.0	35.0	1.8	4.2	4.2
AAM30	18.4	38.0	44.5	2.7	4.9	5.5
AAM50	44.5	60.0	63.0	3.8	5.8	6.8
